# Minimum visual acuity: a new cone specific clinical test

**DOI:** 10.1038/s41433-024-03377-7

**Published:** 2024-09-27

**Authors:** Jeff Rabin, Katelyn Goodroe, Alyssa Hood, Cara Duka, Kyle Dunmon, Darien Bouaphavong, Thinh Truong

**Affiliations:** University of the Incarnate Word Rosenberg School of Optometry, San Antonio, TX USA

**Keywords:** Outcomes research, Biotechnology

Visual acuity (VA), the cornerstone of eyecare, can yield symptoms despite 20/20 [1]. Optimal VA is 20/10 based on cone spacing. Vernier acuity (minimum misalignment) is 3” arc, 10x < cone spacing, a *hyperacuity* mediated by visual cortex [2–4]. Minimum visible acuity [2] (MVA, thinnest black line detectable on white background) is 1” arc. Hence MVA (pilot avoiding fine wire), is a potential hyperacuity never used clinically. We describe a cone specific test of MVA, including monocular, binocular, chromatic, and orientation specific performance.

**Fig. 1:**
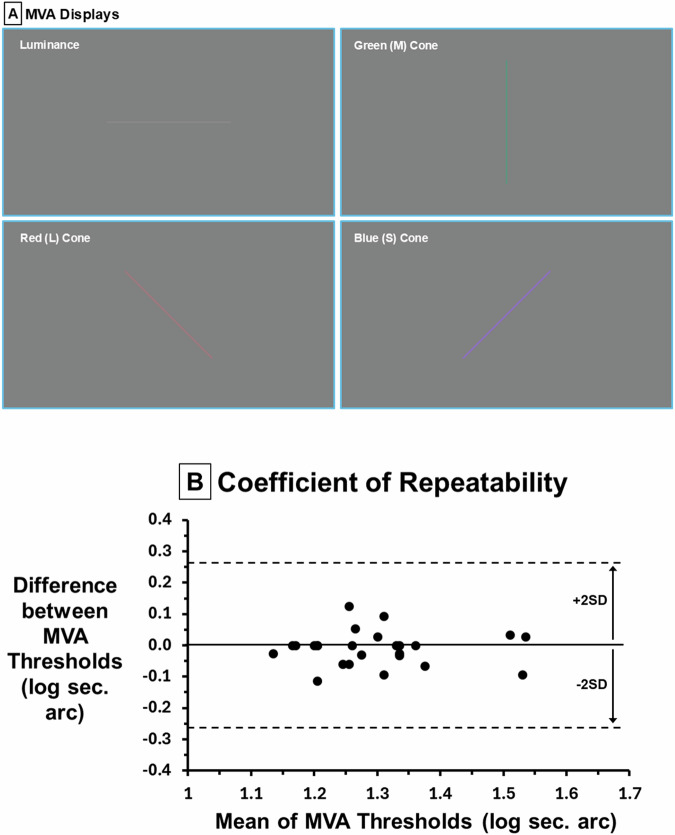
MVA displays and coefficient of repeatability. **A** MVA Displays. MVA maximum thickness stimuli shown for luminance (grey, upper left), L cone (red-orange, lower left), M cone (green, upper right), and S cone (purple, lower right). On each trial a single line with specific color and orientation appeared. Subjects reported both orientation and color. Sixteen stimuli appeared at each thickness level which varied in 0.16 log steps affording a precision of .01 log units per trial. **B** Coefficient of repeatability (COR). The COR is the 95% confidence interval for within patient change, critical for a new test. For a single patient, values falling outside the COR on a second measure indicates significant change. To estimate the COR, the Bland Altman plot shows the difference between right and left eyes (across all colors) plotted against their respective means. The mean difference was zero indicating no bias (between right and left eyes) and 2 SD above and below the mean difference is the COR, which is 0.3 or only 2” of arc indicating a repeatable test.

Twenty-five healthy young adults (VA ≥ 20/30, mean age ± SD: 26 ± 3, 13 females) participated in our IRB approved protocol after written informed consent. MVA stimuli were 1.4° vertical, horizontal, or oblique (45°, 135°) lines centered on a Microsoft Surface display (3.7° x 2.1°) at 4 m in a dark room. Each line was an increase in red (L), green (M), blue (S) cone or luminance (grey) against a grey background (24.7 cd/m^2^, *x, y* = 0.318, 0.355) limiting stimulation to each pathway based on chromaticity and luminance [5]. Contrasts 16%: L, M, luminance; 128%: S cones due to sparse distribution and lower CS [5]. Line thicknesses varied from 60” to 10” arc in 0.16 log steps (16 stimuli/thickness level, 4 orientations, 4 colors). Each trial consisted of a single line centered on the display wherein subjects identified stimulus orientation and color (0.01 log units/trial, Fig. 1a). Contrast, orientation, eye tested were randomized across trials. Binocular followed monocular to minimize learning. Monocular (*P* > 0.18) and binocular (*P* > 0.47) MVA log thresholds were distributed normally (Jarque–Berra test). Repeated-measures ANOVA, paired t-tests (Bonferroni correction) and Bland-Altman were used for analyses.

**Fig. 2:**
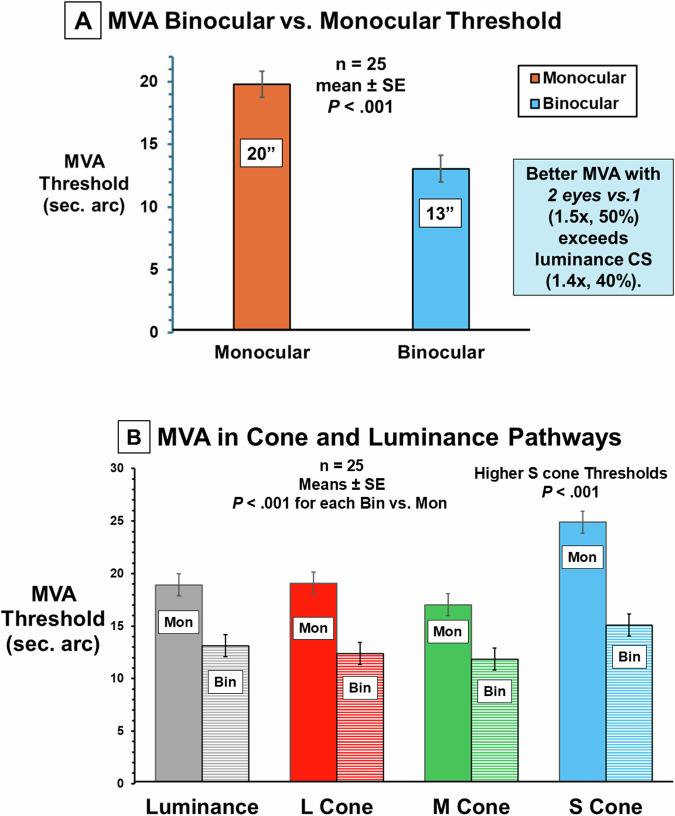
Binocular enhancement of MVA and cone specific MVA. **A** Binocular Enhancement. Mean (±1SE, across all colors) monocular and binocular MVA thresholds (sec. of arc) show a significantly lower binocular threshold (50%, higher sensitivity with two eyes vs. one) than the classical finding of 40% for contrast sensitivity. **B** Cone Specific MVA. Mean (±1SE) monocular and binocular MVA thresholds for luminance, L, M, and S cone pathways showing binocular enhancement (lower binocular thresholds for each pathway) with significantly higher thresholds for S cones attributable to their sparse distribution and lower contrast sensitivity [5].

Repeated measures ANOVA across eye tested and cone type showed significant effects of eye (F = 15.5, *P* < 0.001) and cone type (F = 50.8, *P* < 0.001). Since MVA was not different between right and left eyes (*P* > 0.73) means were used for monocular analyses. The coefficient of repeatability, 95% confidence interval (CI) for within-patient change, was 0.3 log thickness (2” arc, Bland Altman, Fig. 1b). Across all colors, mean binocular threshold (1.12 log s, 13”) was significantly lower than monocular (1.30, 20”, mean difference 0.18 log s, 95% CI 0.16–0.20, *P* < 0.001, Fig. 2a). There was no difference between L, M and grey MVA thresholds (*P* > 0.32) while both S cone monocular MVA (1.40) was reduced vs. L, M, luminance (1.26, mean difference 0.14, 95% CI 0.09–0.17, *P* < 0.001) as was S cone binocular MVA (difference 0.08, 95% CI 0.04–0.13, *P* < 0.001, Fig. 2b). Binocular S cone MVA was decreased for oblique vs. horizontal/vertical (*P* < 0.02). Binocular L cone MVA was improved for horizontal compared to vertical (*P* < 0.001).

MVA binocular and orientation effects support cortical processing. Decreased S cone MVA reflects the paucity of S cones. 50% enhancement of binocular vs. monocular MVA exceeds 40% for CS indicating binocular cortical processing. Improved L cone binocular horizontal MVA may increase disparity enhancing stereoacuity. MVA shows promise for disease detection/monitoring, occupational application, and as an outcome for gene therapy. Ongoing research is assessing age, orientation, and color naming. Preliminary results show high sensitivity for detection of color deficiency and improvement with color correcting lenses.
